# Form and Function of the Vertebrate and Invertebrate Blood-Brain Barriers

**DOI:** 10.3390/ijms222212111

**Published:** 2021-11-09

**Authors:** Alicia D. Dunton, Torben Göpel, Dao H. Ho, Warren Burggren

**Affiliations:** 1 Developmental Integrative Biology Group, Department of Biological Sciences, University of North Texas, Denton, TX 76203, USA; torben.goepel@unt.edu (T.G.); warren.burggren@unt.edu (W.B.); 2 Department of Clinical Investigation, Tripler Army Medical Center, Honolulu, HI 96859, USA; dao.h.ho.civ@mail.mil

**Keywords:** blood-brain barrier, morphology, evolution, neurovascular unit, nervous system

## Abstract

The need to protect neural tissue from toxins or other substances is as old as neural tissue itself. Early recognition of this need has led to more than a century of investigation of the blood-brain barrier (BBB). Many aspects of this important neuroprotective barrier have now been well established, including its cellular architecture and barrier and transport functions. Unsurprisingly, most research has had a human orientation, using mammalian and other animal models to develop translational research findings. However, cell layers forming a barrier between vascular spaces and neural tissues are found broadly throughout the invertebrates as well as in all vertebrates. Unfortunately, previous scenarios for the evolution of the BBB typically adopt a classic, now discredited ‘*scala naturae*’ approach, which inaccurately describes a putative evolutionary progression of the mammalian BBB from simple invertebrates to mammals. In fact, BBB-like structures have evolved independently numerous times, complicating simplistic views of the evolution of the BBB as a linear process. Here, we review BBBs in their various forms in both invertebrates and vertebrates, with an emphasis on the function, evolution, and conditional relevance of popular animal models such as the fruit fly and the zebrafish to mammalian BBB research.

## 1. Introduction

### 1.1. Goals of This Review

The current textbook definition of the blood-brain barrier (BBB) typically reads something such as: “a physical barrier made of endothelial cells that line capillaries of the brain; it provides a semi-permeable barrier movement of certain substances into and out of the brain to maintain homeostasis of the central nervous system”. Although this description of the BBB is generally accurate, there are important caveats and nuances of the BBB that are not captured by this definition. Firstly, this definition is species-agnostic, but the implication is that the description of ‘the BBB’ is for a mammal, or more specifically, a human. Secondly, this definition provides little information with respect to how the BBB evolved to become what it is today across taxa. Thirdly, this definition implies that there is a single type of blood-brain barrier. To a comparative physiologist and an evolutionary biologist, understanding the origins of the BBB in its various iterations is crucial to understanding its form and function as it exists today.

Studies on the BBB have been dominated by human health and medicine, with little focus on the evolutionary and ecological relevance of the BBB. Consequently, in this review, we will report not only what is currently known of the BBB in humans and their animal models, but broadly across numerous taxa. Additionally, we explore gaps and limitations of our current knowledge in this field to encourage wider and deeper understanding of the role of the BBB in physiology. We review current as well as historic literature on the BBB beyond the human-centric focus, across several taxa, including mammals, birds, fish, amphibians, reptiles, and invertebrates. In each section, we primarily discuss the function of the barrier, with a sufficient description of the structure and its heterogeneity among groups to understand functional differences. We also discuss the need for an ecological and evolutionary framework and indicate new directions for future experiments in the field.

### 1.2. The Historical and Current Focus on BBB of Humans

Why has our understanding of blood-brain barriers been largely human-centric, and why does it still continue to be so today? The answer may lie in both the momentum gained from its discovery and its critical role in neurodegenerative disorders. The earliest recorded demonstration of the existence of the BBB occurred in the context of laboratory experimentation to further understanding of human medicine by the German physician Paul Ehrlich, when, while studying chemotherapeutic agents, he inadvertently observed that dyes such as trypan blue injected systemically into the adult mouse were excluded from the brain and spinal cord [1]. This led to his Nobel-prize-winning work that resulted in the chemotherapy drug used to treat syphilis. From this early observation, a burst of experiments was conducted to identify the morphological, physiological, and biochemical properties of the BBB and to determine in which organisms it exists [2,3,4,5,6,7,8,9]. These experiments were driven less by interest in the general biology of the nervous system than by a desire to better understand the role of the BBB in human health and disease.

From studies of model animals (including the fruit fly *Drosophila*, birds, fish, and mammals), it is now very well established that the BBB limits the passage of substances from circulation into the central nervous system (CNS) [10]. Additionally, the BBB serves as a gatekeeper to the neural tissues of the brain by regulating the passage of essential amino acids, ions, and metabolites necessary for proper function of the CNS [10]. The interest in BBB function in human health and disease is at the forefront of neuroscience, due to the key role that the BBB plays in the onset of several diseases such as Alzheimer’s disease [11]. More recently, the mammalian BBB has been studied for the purpose of optimizing therapeutic drug-delivery into the CNS, where the goal is to hijack existing transport mechanisms of the BBB to allow selective entry of therapeutic molecules, while minimizing the ill effects of a leakier BBB [12,13,14]. Current research has focused mainly on pathological conditions; however, this perspective is limited. The CNS not only maintains proper physiological function and survival, but its suboptimal function is also intrinsically linked to an animal’s behavior and fitness. Though the focus of the BBB has been mainly on humans, the critical ecological and evolutionary function that it plays is overlooked in the majority of the literature.

### 1.3. Evolution of Blood-Brain Barriers

The evolution of the blood-brain barrier has been addressed in recent papers [15,16], but rarely has this topic been a focal point of discussion. It has been proposed that the main function leading to the evolution of the BBB is the maintenance of neuronal environment for proper signaling [15]. Additionally, it is suggested that the selection pressures for a BBB increase with the increasing complexity of the CNS [17]. The BBB is conserved across many taxa, so it is essential to further examine its importance at the level of individual animals and the stressors that they face, as well as how an individual’s response may contribute to the fitness of a population and its evolution. Many intrinsic and extrinsic factors influencing animals cause changes in blood composition. Importantly, this places the BBB at the interface of the CNS and environment. It also produces strong selective pressures upon the BBB. Understanding both how the BBB has evolved and how the BBB responds to current conditions provides valuable insight into how the environment has shaped the evolution of this nervous system interface.

The evolution of complex nervous systems with centralized processing structures, i.e., brains, has led to the evolution of BBBs to provide functions such as ion homeostasis and protection from xenobiotics. While most vertebrate BBBs fundamentally involve the vascular endothelium, invertebrate barriers generally consist of glial cells rather than vascular endothelium (which they often lack). Consequently, it has been proposed that the glial BBB represents the plesiomorphic state, often referred to as “primitive” [15]. The coexistence of a glial and pericyte BBB in cephalopods has been interpreted as a transitional state towards the state of the “highest” organization in the form of an endothelial BBB [18,19]. Unfortunately, this well-accepted linear model of BBB evolution depends on a *scala naturae* interpretation of evolution and conflicts with the modern phylogenetic view. Put differently, the oversimplified evolutionary sequence “insect --> cephalopod --> vertebrate” often espoused in reviews of the blood-brain barrier simply cannot be upheld in modern phylogenetic approaches to evolution.

**Figure 1:**
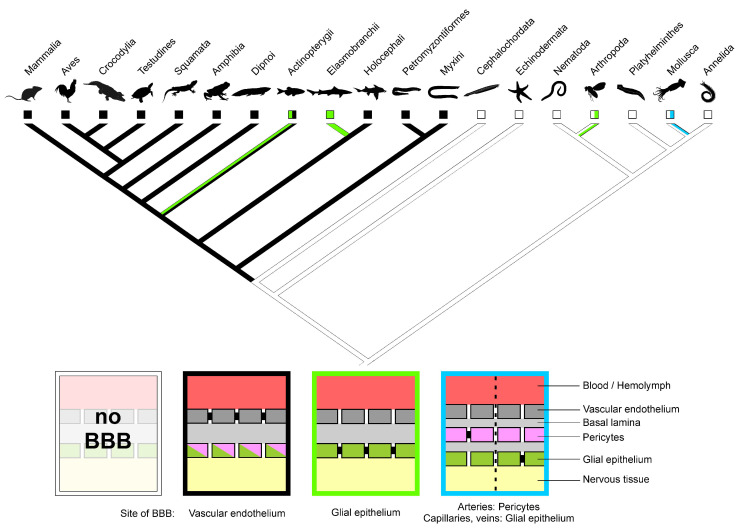
Evolution of blood-brain barriers in Bilateria. Parsimony-based reconstruction of ancestral states shows BBB at the vascular endothelium as apomorphy of vertebrates; glial BBBs have evolved multiple times independently. Boxes show simplified depictions of the BBB; localization of the BBB is indicated by black occlusions between cells. Box color refers to character state in the cladogram. Phylogeny simplified after [27,28,29,30]; most invertebrate taxa without a BBB have been left out for sake of simplification. Ancestral state reconstruction was performed using Mesquite 3.61 [31] silhouette images courtesy of phylopic.org.

Within vertebrates, elasmobranchs and sturgeons exhibit a glial BBB [20]. Jawless fish (hagfish and lamprey), chimaeras, bichirs, and all teleosts, as well as all sarcopterygians, possess an endothelial BBB [6,20] (Figure 1). Based on the linear model of BBB evolution from glial to endothelial, and the interpretation of elasmobranchs and sturgeons as representatives of plesiomorphic morphology, Bundgaard and Abbott (2008) hypothesized a glial BBB in the ancestor of vertebrates. This hypothesis, however, requires assumptions of multiple independent origins of the endothelial BBB in vertebrates and is further explained by the presence of glial BBBs in the unrelated arthropods and cephalopods [20]. Nonetheless, parsimony-based reconstruction of the evolution of blood-brain barriers in triploblastic animals, i.e., Bilateria, paints a distinctly different picture (Figure 1). Blood-brain barriers must have, in fact, evolved independently multiple times: (1) within Mollusca, (2) within Arthropoda (likely at least twice: within arachnids and crustaceans/insects respectively), and (3) within vertebrates. The involvement of different tissues (endothelia, pericytes, glia) as well as the separate evolutionary origin (Figure 1) favors interpretation in the different taxa of BBBs that convergently evolved. Even the glial barriers found in cephalopods (in capillaries and veins), arthropods, elasmobranchs, and sturgeons have evolved independently. Thus, the proposed idea of the general evolutionary direction from a glial BBB towards an endothelial BBB requires re-evaluation as no single such transformation can be assumed based on ancestral state reconstruction (Figure 1). Moreover, evolutionary transformation in the “reverse” direction from an endothelial BBB to a glial BBB has to be assumed in at least two taxa of fish (Elasmobranchii and Acipenseriformes; Figure 1; e.g., [21]) as an endothelial BBB has to be assumed as the character state at the origin of vertebrates (Figure 1). Why sturgeons as well as sharks, rays, and skates have evolved a glial BBB, however, remains unclear. One possibility might be that adaptation and thus evolutionary transformation of the vascular endothelium towards a different role brought along a loss of barrier function, which then had to be compensated for by the glial tissue.

The phylogenetically distant occurrence and ultrastructural details of blood-brain barriers [22] allow for the assumption that both vascular endothelium and glia have the potential to form restrictive barriers [15,21]. Increased signal processing in the brain resulting from more complex sensory input and/or more elaborate cognitive and behavioral capabilities created a selection pressure for a diffusion barrier around the brain. This favored the opportunistic evolution of the barrier-forming capabilities of the tissues presently found in blood-brain barriers [15,19,21].

Despite the independent evolution of BBBs, deep homology [23] of subcellular components, such as transcellular transport systems shared across invertebrates and vertebrates (e.g., [24,25,26]), allows for the use of invertebrate model systems to draw implications on vertebrate (particularly human) BBB function.

We now turn to detailed description of the blood-brain barriers of various taxa. Having criticized the typical ‘*scala naturae*’ interpretation taken by most investigators, we start with the mammals and work ‘down’ into the invertebrates. We note the irony of our taxonomic approach and emphasize again that the BBB independently evolved numerous times, and so the order of which we discuss the various taxa does not imply an evolutionary progression of the blood-brain barrier.

## 2. The Blood-Brain Barrier of Mammals

Since Paul Ehrlich ‘s discovery of the blood-brain barrier, the mammalian BBB has been intensely studied in humans, rodents, rabbits, pigs, and dogs [32]. Despite the vast number of publications on the mammalian BBB, the subjects under examination remain exclusively restricted to “model” mammals that serve to better understand human health, disease, and therapeutics, with little regard for the ecological relevance. In this section, we aim to highlight the main features of mammalian BBB and present what is currently known about the mammalian BBB within an ecological, comparative context. We will discuss the differences in BBB structure and function among mammals and explore gaps in knowledge and special considerations needed to advance our understanding of the mammalian BBB beyond human health and disease.

### 2.1. Form and Function of the Mammalian Blood-Brain Barrier

#### 2.1.1. Form

Early experiments assessing the pharmacological effects of compounds injected intravenously versus intraventricularly in the mouse [33] proposed that a physical barrier existed between the brain and the systemic circulation. This hypothesis was later supported by the inability of albumin-bound proteins to enter the brain [34]. Additional studies performed on dogs and rabbits confirmed these early observations [7,8].

With the advent of the electron microscope, researchers were able to visualize and confirm the ultrastructural features of the mammalian BBB during the mid-1950s into the late 1980s, primarily in mice and rats [32]. It was determined through these studies that the mammalian BBB consisted of specialized endothelial cells that line the cerebral capillaries. The endothelial cells express membrane proteins called tight junction proteins (occludin, claudins, cingulin, and membrane-associated guanylate kinase proteins) and adhesion molecules (cadherins, platelet endothelial cell adhesion molecule, and junctional adhesion molecules) that regulate the selective paracellular diffusion of molecules into and out of the CNS [32,35].

#### 2.1.2. Function

**Figure 2:**
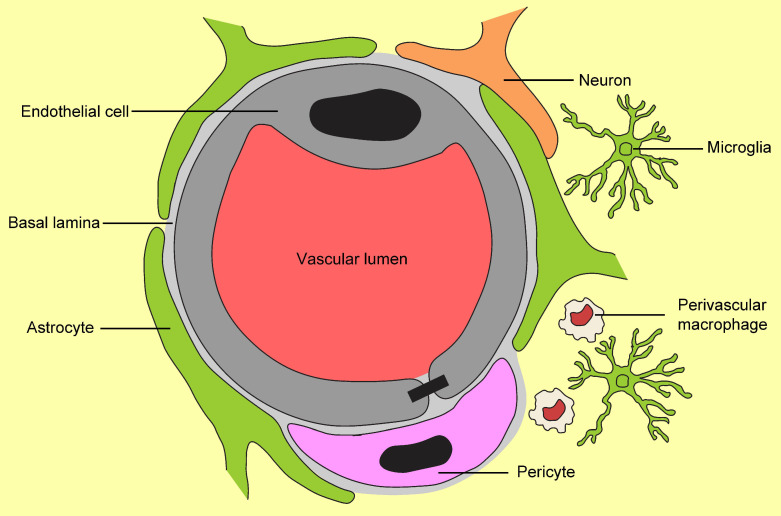
Schematic representation of the blood-brain barrier in mammals in the context of the neurovascular unit. Cross-section of a mammalian BBB with associated components of the neurovascular unit (NVU). Mammals have an endothelial barrier with tight junctions (black bar) between the endothelial cells. The function of the BBB depends upon the synergistic interaction of the many cellular components that make up the NVU. Astrocytes, pericytes, neurons, microglia, and perivascular macrophages can modulate the permeability of the BBB via direct contact and/or via release of paracrine factors such as cytokines. Image adapted from [36,37].

It has long been established that the function of the mammalian BBB is to regulate the passage of compounds and substances into and out of the CNS in order to maintain homeostatic balance. Trafficking of compounds between circulation and the brain occurs at the endothelial cells of cerebral blood vessels; however, other components that interact with the brain’s microvascular endothelial cells to modulate BBB function in mammals include endothelial cells, pericytes, astrocytes, microglia, perivascular macrophages, the basal lamina, and neuronal cells (See Figure 2; adapted from [36,37]). Collectively, the interaction of these components makes up what is known as the functional neurovascular unit (NVU; [38]). The NVU tightly regulates the local milieu in the brain and CNS. Selective paracellular transport of potassium (K+) and sodium (Na^+^) across the BBB is maintained by tight junction proteins, while the transcellular transport of ions, water, and small polar molecules are through passive diffusion, efflux pumps, ion/water channels, and active transporters [35]. Active transport of compounds out of the brain is carried out by efflux transporters in the endothelial cells. Efflux transporters include the glucose transporter 1 (GLUT1), P-glycoprotein (P-gp), breast cancer resistance protein (BRCP-1), organic anion transporting polypeptide (OATP), and G protein-coupled receptors (GPCRs; [39,40].

In all mammals, there is phenotypic heterogeneity in the BBB with respect to the brain region [41]. Specialized areas of the brain such as the hypothalamic region have an incomplete BBB that is highly fenestrated with less tight junctions, creating a more permeable barrier [42]. These brain regions that are surrounded by more permeable BBB play a large role in metabolism, selectively allowing hormones and nutrients to cross the BBB. In particular, satiety signals such as neuropeptide Y (NPY), leptin and ghrelin, and insulin enter at these sites to regulate food intake and overall energy homeostasis. Additionally, the BBB surrounding the circumventricular organs (CVO) allows for the passage of hormones into the bloodstream, all the while freely sensing compounds in the bloodstream [39,43].

### 2.2. Factors Altering BBB Function

#### 2.2.1. Development

In mammals, development of the BBB can be divided into three general phases—angiogenesis, differentiation, and maturation—with the timing of each phase occurring in a species-dependent manner (reviewed in [41]). The first two phases of BBB development occur in late gestation in rodents and humans (third trimester), with the BBB being fully functional and maturing from this point forward [44,45,46].

The function of the BBB in developing mammals has been extensively investigated in humans, rodents (mice, rats, guinea pigs), and other mammals to demonstrate the BBB’s ability to exclude horseradish peroxidase and trypan blue from the brain in very early stages of the developing embryo. These studies solidify the position that developing mammals possess fully functional BBBs (thoroughly reviewed in [47,48,49]). For example, in the human fetus, as early as 8 weeks’ gestation, the BBB can protect the brain when the placenta fails to prevent noxious substances from entering the fetal circulation [50]. This early protection of the brain/CNS is also evident in fetal mice, where the BBB is able to exclude maternally ingested nanoparticles from the fetal brain as early as day 12–19 of gestation [51].

#### 2.2.2. Nutrition

The role of nutrition in mammalian BBB function has been characterized in the context of human health, with little consideration of the ecological significance of processes in non-human mammals under examination [17,52]. The majority of studies in nutritional effects on the mammalian BBB have been performed in mice, where malnutrition in the form of high fat food intake impairs BBB function through a decrease in tight junction proteins. In turn, this causes neuroinflammation via increased reactive oxygen species (ROS), cytokines, and an increased influx of peripheral immune cells into the brain, increased hippocampal albumin content [53], and increased passage of sucrose and albumin across the BBB of the hippocampus and hypothalamus [54]. Additionally, obesity in mice reduces insulin transport across the BBB due to impairment of insulin transporters, which is reversed by subsequent starvation [55]. The ability of mouse brain microvascular endothelial cells to counter the BBB-damaging effects of starvation comes in the form of autophagy to reduce ROS and prevent the redistribution of tight junction protein, CLDN5 [56]. Whether this mechanism is specific to mice (*Mus musculus*), and whether this has important implications for metabolic processes in wild mice, remains to be investigated.

A comparison of human and rat BBB function reveals species-specific discrepancies in glucose handling by the BBB. In humans, a short bout of starvation induces an increase in glucose transport across the BBB into the brain despite decreased plasma glucose levels, suggesting preferential permeability of the BBB to glucose to conserve brain function [57]. In rats, however, it appears that drastic changes in plasma glucose levels may not significantly alter glucose transport across the BBB [58].

In the case of hibernating mammals, BBB function is modified to help prevent injury during starvation and hypothermia. As mammals enter hibernation, the expression of the ketone transporter monocarboxylic acid transporter 1 is upregulated in the BBB [59]. This is unique to hibernators such as the ground squirrel (*Spermophilus tridecemlineatus*) and is not observed in rats undergoing induced hypothermia [59]. In chipmunks (*Tamius sibiricus*), leptin, thyroxine, and hibernation protein complex (HPc) are actively transported across the BBB during hibernation bouts, suggesting an adaptive role of the BBB to maintain and induce hibernation in these animals [60,61]. However, the mechanisms that alter BBB function during hibernation remain unclear.

#### 2.2.3. Hypoxia

The effects of hypoxia on the mammalian BBB have primarily been examined in humans in the context of either high altitude exposure (mountain sickness) or ischemia induced by disorders such as stroke, cardiac arrest, and respiratory distress [62,63]. Moreover, most studies report the effects of artificially induced hypoxia in laboratory rodents, with the assumption that the results can be generalized to all mammals [64,65]. Chronic exposure to low oxygen levels in mice causes the BBB to become leaky, and the depletion of microglia further exacerbates the hypoxia-induced leakiness [66]. Furthermore, unwanted angiogenesis occurs with mild hypoxia in mice [66]. Interestingly, in Wistar rats, administration of exogenous ghrelin reverses the reduction in tight junction proteins (occludin) caused by chronic hypoxia, suggesting that ghrelin acts to reduce inflammation, ROS, and oxidative stress caused by hypoxia [67,68].

#### 2.2.4. Ambient Temperature

Extreme temperatures affect the BBB permeability, with an association between hyperthermia and BBB disruption in rats [69,70]. In the case of hypothermia, artificial induction in rats, as well as natural hibernation in ground squirrels, increase BBB permeability [71]. The mechanisms and consequences of increased BBB permeability during the lowered body temperatures caused by hibernation and torpor remain poorly understood. However, we speculate that the consequences are quite different from non-hibernating mammals [72].

#### 2.2.5. Psycho-Social Stress

Psycho-social stress modulates the mammalian BBB via mechanisms associated with inflammation and oxidative stress [52,73]. In mice that are susceptible to stress, social defeat caused increased leakiness of the BBB through inflammation, oxidative stress, and extracellular remodeling associated with microglial activity [74]. Social isolation in young rats resulted in BBB disruption characterized by a dramatic increase in inflammatory cytokine Interleukin-6 (IL-6), and a subsequent increase in the oxidative stress marker NOX2 in the brain [75]. Additionally, acute restraint stress in rats cause increased permeability of the BBB through mast cell-mediated inflammation [76].

#### 2.2.6. BBB and Developmental Malleability

Environmental stressors alter the maturing BBB in ways that can permanently alter the adult BBB. The BBB is a highly dynamic structure that alters the exchange of molecules between the bloodstream and the brain in response to homeostatic adjustments in health and disease. Across the lifespan, BBB permeability adjusts to continuously changing physiological states [52]. For example, maternal obesity increases BBB permeability in the offspring [77], leading to higher exposure to leptin and ghrelin, causing alterations in metabolic control in adulthood. At perinatal stages, the expression of tight junction proteins (claudin-5 and occludin) and extracellular matrix components (laminin and collagen IV) is higher than in adults, whereas the expression of the pericytic marker platelet-derived growth factor receptor beta (PDGFRβ) increases gradually over time. These molecular differences between the developing and adult NVU might account, for instance, for the well-preserved BBB integrity in neonatal rats after acute stroke compared to vascular injury in adults [52]. Fetal brain injury due to hypoxic-ischemia increases the transfer of interleukin-1beta (IL-1B) across fetal BBB in sheep due to BBB dysfunction, likely via an IL-1B-specific transporter [78,79,80]. Additionally, cytokine IL-6 has also been shown to play a primary role in BBB permeability via the disruption of tight junction proteins in fetal sheep suffering from brain ischemia [81].

#### 2.2.7. Environmental Toxicants and Pollutants

The effects of environmental toxicants on the mammalian brain have been widely studied. However, investigation of the impact of environmental contaminants on the function of BBB of wild mammals is lagging [82,83]. The majority of studies describe direct effects of environmental toxicants on the BBB in isolated brain capillaries of laboratory rodents exposed to toxicants or in vitro mammalian cell models of the BBB. In isolated brain capillaries of rats exposed to tetrachlorodibenzo-p-dioxin (TCDD), there is significantly increased activity and expression of P-glycoprotein [84], while brain capillaries of mice exposed to polychlorinated biphenyls (PCBs) have deranged expression of tight junction proteins associated with increased BBB permeability [85]. Additionally, mice exposed to pollutants in vehicle emissions show increased BBB permeability along with decreased tight junction protein levels [86].

Stray dogs in highly polluted areas of Mexico show BBB dysfunction compared to those living in less-polluted areas [87]. Although most toxicants readily cross the BBB, there is evidence that the BBB does actively regulate entry of some perfluoroalkyl acids (PFAAs) into the brains of polar bears in east Greenland [88].

### 2.3. Special Considerations

Outside of human BBB physiology and rodent models, very little has been investigated in “non-traditional” mammals. The elucidation of mammalian BBB function has been centered on human health and disease; as a consequence, there is a lack of information on the form and function of other mammals, especially in an ecological/comparative context. The importance of the BBB in regulation of circadian rhythms, metabolic control, as well as the effects of hypoxia, temperature, and nutrition on the BBB thus argues for further exploration of the BBB in hibernating mammals (e.g., bears, ground squirrels, etc.), high-altitude mammals, burrowing mammals, and aquatic/diving mammals.

Very few truly comparative studies exist for the mammalian BBB. These scarce but valuable comparative studies highlight the need for fuller understanding of species-specific functions of the BBB. A recent comparative study has involved isolated and cultured brain microvascular endothelial cells of mice, rats, and pigs [89]. All cells expressed efflux transporters, P-glycoprotein and transferrin transporters, and tight junction proteins. However, the expression of the tight junction protein ZO-2, induced by the presence of astrocytes, was species-dependent. Additionally, the BBB integrity as measured by trans-endothelial electrical resistance (TEER) was different among the cells of the different species.

### 2.4. Knowledge Gaps/Limitations

Several key questions arise from the paucity of ecologically relevant studies of the BBB in mammals. These and other questions should be answered to better understand the mammalian BBB in its entirety:

- What are the unique characteristics of BBBs of mammals that inhabit different environments (e.g., aquatic versus terrestrial, high-altitude versus sea level, cold versus hot)?
- How does the form and function of the BBB of wild mammals differ from that of laboratory mammals?
- What are the implications of the behavior on BBB function in mammals, especially characteristics associated with social versus non-social animals?
- What is the impact of parental strategies and maternal care on mammalian BBB (humans versus marsupials versus rodents)?
- What is the impact of environmental contaminants on the BBB of mammals that inhabit polluted areas?
- What are the factors that influence the development of the mammalian BBB across species?

## 3. The Blood-Brain Barrier of Birds

### 3.1. Form and Function of the Avian Blood-Brain Barrier

Historically, interest in the BBB of birds was due to the ease of observation of the developing BBB *in ovo*. Thus, the structural and functional development of the BBB of birds, namely the domesticated chicken, has been intensely studied, beginning in the early 1970s [90] and continuing into the mid-1990s ([47]). As with mammals, birds possess an endothelial BBB wherein tight junctions between endothelial cells that line cerebral vessels form a selective barrier between the brain/CNS and the circulating blood [91,92,93]. Structurally, the NVU of birds is organized similarly to mammals with neurons, astrocytes, pericytes, and microglia in direct communication with the basement membrane and capillary endothelial cells. Of particular interest is the role of astrocytes of the NVU in long-distance flight. During long-distance flight in the semipalmated sandpiper *Calidris pusilla*, the astrocytes number is reduced and the balance between Type I and Type II astrocyte populations is altered [94]. It remains to be seen how these changes impact BBB function and migratory behavior. Additionally, the involvement of astrocytes in the development and maintenance of the BBB is crucial in birds [95], similar to mammals [16]. A study detailing the histochemical and ultrastructural features of the developing and mature BBB in both the chicken and the quail identified differences in alkaline phosphatase and butyryl cholinesterase expression as well as the function of the BBB between chicken embryos and quail embryos and hatchlings [96]. How these differences affect the BBB function remains unclear.

### 3.2. Unique Features of the Avian Blood-Brain Barrier

Compared to mammals, very little is known of the BBB function in birds. It is often assumed that since mammalian and avian BBBs are similar in form, the function is similar also. This may often be the case, but there is evidence that point to BBB functions that are unique to birds. For example, one notable difference between birds and mammals is the existence of the glycogen body (*corpus gelatinosum*) in birds. The glycogen body is located in the rhomboidal sinus of the lumbosacral region of the spinal cord, and is surrounded by a functional BBB, which has been proposed to maintain homeostatic balance of the glycogen body [97,98]. Although the exact function of the glycogen body is not known, it has been hypothesized that it plays a role in insulin handling, glucose homeostasis, and osmoregulation [97,98,99]. This suggests a unique role of the BBB in metabolic control in birds.

Another unique characteristic of the BBB of birds is the relatively high permeability to serotonin from the periphery, not observed in mammals [100]. In quail (*Coturnix japonica*), serotonin administered systemically elicited changes in behaviors that are centrally regulated, including feather bristling, crouching, eye closure, and sleep-like behavior [100], all suggesting a distinct role of the BBB in regulating behavior of birds.

Ghrelin transport into the brain is tightly regulated by the BBB of both birds [101] and mammals [102,103]. However, in addition to the traditional role of ghrelin in the regulation of food intake [104], ghrelin regulates migratory behavior in the garden warbler (*Sylvia borin*) [105]. Furthermore, migration in birds such as garden warblers and whitethroats (*Sylvia communis*) cause significant increase in ROS and oxidative stress in the brain [106], suggesting a potential disruption of the BBB, leading to migration-driven physiological changes in birds.

### 3.3. Factors Altering the Avian Blood-Brain Barrier

#### 3.3.1. Nutrition

A decline in wild bird populations throughout the northern hemisphere over the last quarter century has been attributed to thiamine (vitamin B1) deficiency [107,108,109]. In domestic and wild birds, thiamine is crucial to proper functioning of the brain and CNS, with deficiency causing abnormal behavior and posture, paralysis, head tremors, and a loss of appetite in adults [107]. Thiamine deficiency is associated with BBB dysfunction and breakdown in mammals [110,111,112,113,114], strongly suggesting a similar consequence in birds. How nutrients can affect the BBB of birds and cause changes in behavior, migration, or neurological function remains largely unexplored.

#### 3.3.2. Temperature

Some species of birds tolerate a wide range of ambient temperatures outside of their normal core temperature of 38–42 °C by undergoing facultative hypo- or hyperthermia [115,116,117,118]. However, very little is known of how ambient temperature and body temperature affect the BBB of birds. To our knowledge, only a single study has examined the direct effect of ambient temperature on the BBB of birds [119]. This study found that the BBB of Huainan partridge chickens (*Gallus gallus*) that were subjected to two weeks of intermittent hyperthermia was damaged and tight junction proteins ZO-1 and occludin were downregulated.

#### 3.3.3. Special Considerations: Migration, Navigation, Altitude, and Attitude (Mating Behavior)

How the BBB of birds is affected by their ecology is largely unknown. Most of the studies in birds have focused on morphological and biochemical aspects of the BBB, while only hinting at the potential functional role of the BBB in their physiology and behavior. For example, we may speculate that the BBB plays an important role in bird navigation given that electromagnetic fields (EMF) have been shown to affect mammalian BBB function [120,121,122,123] and that EMFs disrupt birds’ magnetic compass [124]. Similarly, many questions remain as to whether ambient temperature, psycho-social interactions during mating, high altitude during flight, hibernation, or migration may drive or be driven by changes in avian BBB function.

### 3.4. Knowledge Gaps/Limitations

Below are pertinent questions that need to be answered to better understand the avian BBB:

- Beyond early embryonic and fetal stages of life, how does the BBB of birds differ from mammalian BBB?
- What role does the BBB play in navigation during migration in birds?
- What is the function of the blood barrier that surrounds the glycogen body of birds?
- Does thiamine deficiency, and nutritional deficiency in general, cause behavioral derangement in birds through disruption of the BBB?
- Does the BBB play a significant role in the mating behavior and mating success of birds?
- Can maternally-derived egg yolk factors and eggshell characteristics of oviparous bird species alter the trajectory of BBB development?

## 4. The Blood-Brain Barrier of Reptiles

Compared to the other vertebrate taxa, our knowledge of the BBB and its functions in reptiles (snakes, lizards, turtles and tortoises, and crocodilians) is scant—surprisingly so. Relative to amphibians, which have been used as animal models (see Section 5), and to fish (also used as models for human health and in the context of aquatic and marine contamination, see Section 6), the blood-brain barrier of reptiles has been largely ignored, apart from a few key studies, many now decades old. However, ironically, reptiles nonetheless feature prominently in BBB research—not because of the barrier characteristics of reptiles themselves, but rather for the variety of effects of their venoms (especially snake venoms) on the BBB of mammals [125,126,127,128]. Here, we review literature on the function of the reptile blood-brain barrier, which we recognize reads more like a fragmented, disjointed list of findings rather than a comprehensive account.

### 4.1. Form of the Reptilian Blood-Brain Barrier

As for other vertebrates, the basis of the relative impermeability of the BBB of reptiles is based upon the tight junctions between endothelial cells in the brain capillaries [129,130,131]. Tracer studies in the BBB of the lizard *Anolis carolinensis* indicate relatively complex endothelial cell tight junctions [130]. These tight junctions have a very low permeability that is not easily disrupted, although severe hyperglycemia had the effect of reducing the integrity of endothelial cell tight junctions [130,132]. In addition to the endothelial cells, the reptilian BBB to our current knowledge is made up of pericytes, radial glial cells, and basal lamina [129,133]. The form of the reptilian BBB is hypothesized to more closely resemble that of mammals than that of fish [129], though more study is needed to elucidate this conclusion more clearly.

### 4.2. Factors Altering Reptilian Blood-Brain Barrier Function

As for other vertebrate taxa, toxins have variable effects on BBB integrity, and in some cases, can cross the BBB. For example, the fungicide triadimefon crosses the BBB of the Chinese lizard (*Eremias argus*), entering the neural tissue of the brain [134]. Cadmium crosses the BBB and alters glial structure in the brain of the Italian wall lizard *Podarcis siculus* [133]. Interestingly, in the garter snake *Thamnophis sirtalis*, which may feed on highly toxic *Taricha* newts producing tetrodatoxin (TTX), the neurons of the CNS show no particular resistance to TTX, suggesting active BBB exclusion of this toxin from the neural tissue [135].

### 4.3. Other Characteristics of the Reptile Blood-Brain Barrier

#### 4.3.1. Regeneration

While not having the regenerative capabilities of amphibians, reptiles nonetheless have considerable capacities for regeneration. While the regeneration of gross structures (e.g., limbs, tail) have been studied in reptiles [136], there appears to have been little focus specifically on neural regeneration, and especially of the NVU. In the lizard *Gallotia galloti*, studies of cellular performance following transection of the optic nerve indicate rapid restoration of key components of the neurovascular barrier within a few weeks [137].

#### 4.3.2. Protein Synthesis

The choroid plexus of reptiles actively synthesizes proteins, including lipocalin and transthyretin (prealbumin), which has a high affinity for triiodothyroninie (T3), and to a lesser extent, thyronxine (T4) [138].

#### 4.3.3. Ion Transport and Enzyme Activity

The GLUT-1 isoform of the glucose transporter, widely recognized as a BBB molecular marker, has been characterized in the wall lizard *Podarcis sicula*, where this GLUT-1 transporter occurs in both parenchymal and meningeal vessels [139]. These vessels are typically paired [140], and even adjacent vascular limbs showed highly variable degrees of immunopositivity for GLUT-1, with high GLUT-1 activity in one branch often matched by little-to-no activity in the other.

### 4.4. Knowledge Gap/Limitations

As previously indicated, little is known about the BBB of reptiles—both its structure and its heterogeneity between different reptile taxa—but especially in terms of its functional characteristics. Several key questions arise:

- What are the similarities and differences between reptiles and other vertebrates, perhaps especially focusing on the similarities of birds as their closest relatives?
- Do those reptiles that generally show systemic regenerative properties also have blood-brain barriers that have high self-repair capabilities?
- What are the functional capacities of the reptile BBB with respect to transport of ions, glucose, and other key materials?
- Given how little is known about tight junctions in the reptilian BBB, how representative are the few data available?
- Given how little is known about ion transport in the reptilian BBB, how representative are the few data available?
- Can the ‘ancient’ extant reptiles—i.e., the crocodilians—tell us about the evolutionary history of the reptilian BBB?

## 5. The Blood-Brain Barrier of Amphibians

### 5.1. Form of the Amphibian Blood-Brain Barrier

The structure of the blood-brain barrier of amphibians came under study soon after the BBB was localized to endothelial cells, perhaps as a result of the frequent use of amphibians and especially various frog species as animal models. Consequently, its structural basis is relatively well understood through studies in several genera from different amphibian families [141,142,143]. In accordance with other vertebrates described thus far, the amphibian NVU is composed of the aforementioned endothelial cells joined by tight junctions, glial cells, pericytes, and basal lamina [143]. However, we are unaware of any systematic investigation of similarities/differences among Amphibia. One notable comparative study of the complexity of the tight junctions of the outer blood–cerebrospinal fluid barrier that included amphibians revealed the following ‘complexity relationship’: chicken > carp = rat > frog [144]. Clearly, BBB complexity does not follow a phylogenetic pattern.

While the structure of the BBB in adult amphibians has been described in some detail and seems to resemble that of other vertebrates, its development through metamorphosis is not well-understood. The development of inter-ependymal pores in the brain ventricles across larval development in *Rana pipiens* has been investigated [145], as has regional development of the BBB in the tail and trunk region of the spinal cord and in the brain of *Xenopus laevis* larvae undergoing metamorphosis [146]. Interestingly, the BBB integrity, as measured by several markers related to thyroxine, was highest in the tail regions of the spinal cord and lowest in the trunk and brain, presumably allowing penetration into the CNS of thyroid hormones involved in metamorphosis in *Xenopus*.

### 5.2. Function of the Amphibian Blood-Brain Barrier

Concurrent with investigation of the morphology of the BBB of amphibians, a flurry of papers focusing on the function of the BBB emerged in the 1960s, 1970s, and 1980s. These papers form the basis of the following discussion, as relatively few functional studies have been published in the last two decades. Of special interest in the early literature was the permeability of the BBB of amphibians compared to mammals. In one of the earliest such studies in the mudpuppy *Necturus maculosus*, the brain endothelium was discovered to be largely impermeable to circulating horse radish peroxidase [147]. The endothelium of telencephalic and subarachnoid microvessels in the grass frog (*Rana temporaria*) are impermeable to lactate [141]. As in mammals, the BBB of amphibians also prevents systematically injected inulin from entering the brain in the mudpuppy (*Necturus* sp.) and both larval and adult tiger salamanders (*Ambystoma tigrinum*) [148]. Similar results are reported for iodine blood-brain permeability in *R. temporaria*. Measurements of sucrose filtration coefficient and osmotic reflection coefficient in single brain microvessels of the frog *R. pipiens* revealed that most water movement across the cerebral microvessels was transcellular rather than pericellular [149].

The very low passive permeability of the BBB of amphibians has been attributed to vast endothelial tight junctions, with an estimated 0.1% of the length of the endothelium being ‘open’ [150]. This creates the high electrical resistance typical of other tight junctions [151], which was suggested to result from the endothelial cells themselves given that at least in the frog brain there is no glial covering of the subarachnoid vessels [143]. Nonetheless, the BBB of amphibians was predicted to have a relatively high permeability to lipid-soluble solutes, though this has not been tested to our knowledge.

### 5.3. Factors Altering Amphibian Blood-Brain Barrier Function

Numerous factors affect the integrity of the microvasculature forming the blood-brain barrier of the amphibian brain.

#### 5.3.1. Hypoxia and Hypothermia

Severe hypoxia and the associated metabolic depression in frogs reduces the integrity of the BBB [152]. Cold injury also increases the permeability of the BBB of amphibians [153], and structural alterations in the central vascular plexuses of frogs (*Rana esculenta* and *Rana temporaria*) are associated with chronically reduced body temperature occurring in winter [154], which could result in altered BBB function.

#### 5.3.2. Hypertonicity

The integrity of the tight junctions of the brain endothelium is degraded by hypertonic solutions of mannitol in the newt *Triturus cristatus carnifex*, as evident by increased permeability to ions of the rare earth lanthanum [155]. This increase in permeability in the amphibian brain affected by hyperosmotic treatment (or cold injury) results from increased intercellular rather than transcellular transport [153].

#### 5.3.3. Hydrostatic Pressure

Amphibians have relatively high permeability blood vessels in systemic and pulmonary tissues in general [156,157,158,159,160]. Consequently, they have well-developed and active lymphatic systems for recovery of vascular filtrate [160,161]. Not surprisingly, then, the bulk flow of plasma across the microvasculature of the amphibian brain can be hydrostatically driven and has been modelled to be somewhat independent of transcytosis, perijunctional gaps, and trans-endothelial channels [149].

#### 5.3.4. Biologically Active Compounds

Changes in brain permeability in the frog *Rana temporaria* can be measured within seconds of insult by a variety of chemical agents, including 5-hydroxytryptamine, bradykinin, ATP, ADP, AMP, phospholipase A2, arachidonic acid, leukotriene C4, oxygen-derived free-radicals, ionophore A23187, and unbound Evans blue dye [152]. Depending upon the induction factor, the decreased permeability can be reversed within a few minutes, as found for inflammatory mediators. Serotonin also reversibly decreases BBB integrity in the frog *Rana temporaria* [162]. In contrast, protamine sulphate, neuraminidase, trypsin, melittin, and snake venoms from *Crotalus durissus terrificus* and *Bothrops atrox* all caused irreversible brain permeability increases, while histamine, epinephrine, putrescine, angiotensin II, vasoactive intestinal polypeptide, substance P, neurotensin, vasopressin, adenosine, PGE2, PGF2α, prostacyclin (PGI2), leukotriene B4, albumin, heparin, plant cytokinins, hyaluronidase, thrombin, and wasp venom had no effect on BBB permeability within 5 min of application [163].

Free oxygen radicals induce rapid, dynamic changes in the electrical resistance of the frog brain vessels and could be a mechanism by which vascular endothelial damage occurs in the brain [164].

#### 5.3.5. Viral Infection

Viral infection with Frog Virus 3 (FV3) increases BBB permeability in *Xenopus laevis*. The mechanism is presumed to involve an inflammatory response [165,166]. Interestingly, reduced BBB integrity in *X. laevis* leads to enhanced viral dissemination in the central nervous system of the larvae but not the adults [165]. Additionally, in *X. laevis*, peritoneal leukocytes infected with FV3 or with *Mycobacterium marinum* cross the blood-brain barrier, as revealed by using fluorescently labelled leukocytes [166].

#### 5.3.6. The Blood-Brain Barrier and Nerve Regeneration

Amphibian neural tissue is renowned for its regenerative abilities [167,168,169,170], and the effect of regeneration on the BBB integrity has been investigated in this context. Two studies from the early 1990s suggest that regeneration does affect the BBB. In the frog *Litoria* (*Hyla*) *moorei*, BBB integrity was reduced during optic nerve regeneration during the period of 1–5 weeks following optic nerve crushing [171]. Somewhat similarly, it has been suggested that during tail regeneration in newts, there is a chronic reduction in BBB effectiveness [172].

### 5.4. Other Characteristics of the Amphibian Blood-Brain Barrier

#### 5.4.1. Protein Synthesis

The choroid plexus of both adult and larval frogs (*Limnodynastes dumerili*) and marine toads (*Bufo marinus*) synthesize proteins, principally lipocalin, as occurs in other vertebrates [173]. Protein synthesis at the BBB continues throughout metamorphosis in these species. Unlike reptiles, birds, and mammals, no transthyretin (preablumin) is synthesized in the BBB [138].

#### 5.4.2. Ion Transport and Enzyme Activity

Alkaline phosphatase activity has been identified in endothelium of the brain vasculature in *Rana esculenta* and *R. pipiens* [174,175]. Alkaline phosphatase activity has been localized to both the luminal and abluminal walls of endothelial capillary cells, while Na^+^/K^+^-ATPase occurs only on the abluminal side. This ‘polarization’ suggests different functions for different sides of the brain microvascular endothelial cells [175]. Alkaline phosphatase distribution patterns in the frog central nervous system differ from those in the rat, but in the frog *R. pipiens* it always occurs in at least one structural component of the BBB [174].

Glucose transporters (e.g., GLUT-1) have been characterized in the brain microvessels of the axolotl *Ambystoma mexicanum*. The distribution of these transporters varies with location between singular or paired vessels, and they are more densely located on the abluminal compared to the luminal surface; however, their efficacy in glucose transport has not been characterized [176].

### 5.5. Knowledge Gap/Limitations

As for many animals, few studies on the BBB of amphibians have been carried out in the ecological/environmental context in which amphibians have evolved and currently live. Thus, the following key questions remain to be answered:

- How does the process of metamorphosis, and the associated apoptosis of the PNS in the trunk, as well as the growth of innervation into the growing limbs, alter the integrity of the neurovascular boundaries?
- What effects do potentially large changes in temperature, salinity, and oxygen have on the effectiveness of the amphibian BBB barrier?
- Does the degree of terrestriality affect neural function and especially the integrity of the amphibian BBB?
- Does the considerable regenerative ability of amphibians translate into greater repair capabilities of an injured BBB?
- What differences, if any, exist between the blood-brain barrier of Anura (frogs and toads), Urodela (salamanders and newts), and Apoda (caecilians)?
- Given how little is known about ion and nutrient transport in the reptilian BBB, how representative are the few data available?

In short, studies of the BBB of amphibians as more than simply models for higher vertebrates are greatly warranted.

## 6. The Blood-Brain Barrier of Fish

The first description of a possible BBB in teleost fish was a 1942 study by Lundquist [177], using roach (*Leuciscus rutilus*), bream (*Abramis brama*), perch (*Perca fluviatilis*), and tench (*Tinca vulgaris*). Through injection of the colored dye, Trypan blue, a barrier structure was hypothesized after brains—but not other tissues—remained unstained. A similar study using several different dyes of varying molecular weights determined that goldfish (*Carrasius auratus*) also possess a BBB that can be impaired mechanically [2]. Additionally, it was determined that the BBB of fish may regulate ion concentrations and transport substances differently than mammals [2]. These early experiments used dye exclusion and electron microscopy to determine the presence of a BBB and its morphological characteristics in multiple fish groups [3,4,5,178,179], as reviewed by [15,143].

Although early studies of the fish BBB focused on multiple fish species, literature from the past 10–20 years has focused almost exclusively on teleosts, and among those primarily zebrafish (*Danio rerio*) [180,181,182,183]. This is due to the zebrafish’s utility as a model organism for humans. Outside human health applications, a small number of studies have examined the BBB of other teleost species for environmentally relevant issues such as pollution and aquaculture [183,184,185]. However, many environmental studies address whether certain compounds cross the BBB rather than how such compounds or environmental conditions impact the BBB structure, physiology, and function, as well as the overall biology of fish.

### 6.1. Form of the Piscine Blood-Brain Barrier

#### 6.1.1. Mature Fish

**Figure 3:**
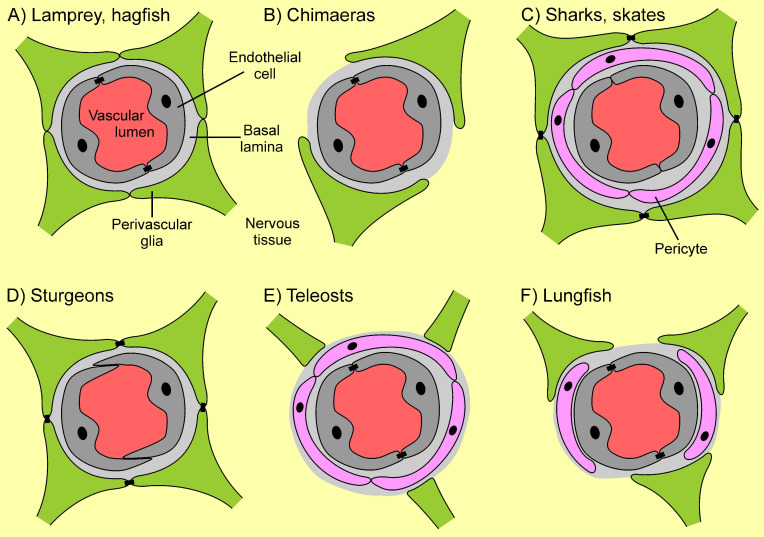
Schematic representation of the blood-brain barrier in fish. Cross section of a fish neurovascular unit; black bars indicate the site of the BBB. (**A**): NVU of lamprey and hagfish. Hagfish/lamprey have an endothelial barrier with tight junctions between endothelial cells. Glial cells form a continuous layer around endothelial cells. Basal lamina is present; no pericytes are present. (**B**): NVU of chimaeras. Chimaeras have an endothelial barrier, basal lamina, and a discontinuous glial layer. Pericytes are not present. (**C**): NVU of elasmobranchs—sharks and skates. Glial cells form the BBB and are joined by tight junction proteins. Endothelial cells are not joined by tight junctions and form a leaky layer. Pericytes are present between glial cells and endothelial cells. Basal lamina is present. (**D**): NVU of sturgeons. Sturgeons have a glial BBB, but glial cells are not joined by tight junctions. Endothelial cells are joined by tight junctions but form a leaky layer. Pericytes are not present; basal lamina is present. (**E**): NVU of teleosts. Teleosts have an endothelial BBB, and endothelial cells are joined by tight junctions. Glial cells are radial glia and are discontinuous. Pericytes are present and are between endothelial cells and glial cells. Basal lamina is present. (**F**): NVU of lungfish. Lungfish have an endothelial BBB, and endothelial cells are joined by tight junctions. Glial cells are present and are discontinuous. Perivascular cells are present, but it is unclear whether these cells are pericytes. Basal lamina is present.

In many fish, the NVU resembles that of other vertebrates, comprising endothelial cells, perivascular glia, pericytes, neurons, and a basement membrane [20,180,186,187]. Within the NVU, there is variation in the location of the BBB, the cell types present, and the abundance of cells [20,178]. This information is summarized in Table 1 and Figure 3. In sturgeons (*Acipenser baerii*, *A. gueldenstaedtii*) and lungfish (*Protopterus annectens*), pericytes such as those seen in other vertebrate BBBs are absent. However, they are present in teleosts as well as in elasmobranchs [20,178,186]. In zebrafish, the BBB is present in all regions of the brain outside the circumventricular organs, pineal gland, and vascular organ of the lamina terminalis [180]. The endothelial cells form capillaries, which are continuous in teleost fish [180]. The lamprey *Lampetra fluviatilis* and the hagfish *Myxine glutinosa* have many tubules and vesicles in their cerebral endothelial cells, distinct from other fish [179,188,189]. Most fish have an endothelial BBB wherein the main barrier is held by endothelial cells joined by continuous tight junctions [10,15,20,143,190]. The tight junction proteins claudin-5 and ZO-1 occur in zebrafish, but less is known about the specific tight junctions which are present in other fish, because earlier studies examined the presence of tight junctions through electron microscopy rather than molecular analysis [3,178,180,182,191]. However, in the brain endothelial cell line (EelB) of the American eel, *Aguilla rostrata*, claudin-5 as well as claudin-3 are present, along with ZO-1 [192]. The presence of claudin-3 indicates that the American eel may have a BBB more similar to mammals, which also have claudin-3 [193]. Therefore, there may be differences in the BBB across teleosts. Notably, a theme present throughout research on the BBB in fish is that zebrafish, while they are considered the “model” teleost in laboratory studies, are not necessarily a representative teleost fish. Therefore, it is essential to consider the diversity of environments inhabited by fish, and that other fish species should be examined to make broad conclusions about piscine blood-brain barriers.

Tracer studies in teleost fish demonstrate that there are compounds such as sulfo-NHS-biotin, Evan’s blue, and horseradish peroxidase (HRP) which cannot pass from the endothelial cells into the brain parenchyma [3,14,180]. Unlike other fish, however, elasmobranchs and sturgeons do not have an endothelial BBB (Figure 3). Rather, they possess a leaky endothelium and a tighter barrier among the perivascular glia [178]. Tracer studies in skates, dogfish, sharks, and rays have demonstrated that dyes are indeed excluded from the brain, but do migrate through the endothelial layer [178]. A similar study in three ancient fish found that sturgeons also exhibit exclusion at the glial level [20]. In elasmobranchs, this glial barrier is joined by tight junctions, while in sturgeons the glial cells are linked by glial lamellae instead, similar to that observed in cuttlefish [20,178].

Glia of the NVU of fishes show some differences from mammalian astrocytes [187]. In contrast to the mammalian BBB, zebrafish do not have astrocytes but radial glia [186,187,194]. Some studies have also identified radial glia as the glial subtype participating in the BBB of elasmobranchs [195]. In other fish, less is known regarding specific glia involved in the BBB and NVU. Therefore, we refer to them here as perivascular glia. Radial glia are neural progenitor cells, which may later develop into other CNS cell types [194,196]. Though morphologically different, they are functionally similar to the mammalian astrocyte and seem to play a similar role in the maintenance of the BBB in zebrafish, including water and ionic regulation [187]. Aquaporin channels are localized throughout the radial glia in zebrafish, facilitating water transport [187].

Additionally, radial glia are thought to contribute to the higher levels of adult neurogenesis observed in teleost fish compared to other vertebrates such as mammals [196]. Areas of neuronal proliferation are present throughout the teleost brain, contrary to the mammalian brain, where these zones are limited to just two brain regions [197]. These neurogenic areas have been studied mainly in zebrafish but have also been identified in other teleosts such as three-spined stickleback (*Gasterosterus aculeatus*) and brown ghost knifefish (*Apteronotus leptorhynchus*) [197,198]. Much less is known regarding neurogenic capacity in other fish, though neurogenic regions were recently discovered in the telencephalon of the catshark, *Scyliorhynus canicula* [197,199].

#### 6.1.2. Developing Fish

The majority of the research examining the development of the BBB has been conducted in zebrafish larvae [182,200,201]. The development of the BBB in zebrafish is thoroughly reviewed by [200]. The BBB begins to develop around 3 days post-fertilization (dpf) [180,182,191,200]. The tight junction proteins claudin-5 and ZO-1 occur in the BBB by 2 dpf, restricting paracellular transport [182,191,200,202]. Dye exclusion studies have shown that at 3 dpf, the BBB restricts some movement of dyes of varying molecular weights into the brain parenchyma [180,182,191,203]. There is varying evidence as to exactly when the different components of the BBB develop. Barrier properties of cerebral microvessels develop as the vessels themselves develop, though all barrier functions may not develop at the same rate [201,203]. In zebrafish, different regions of the brain develop full barrier characteristics at varying timepoints [203]. The hindbrain becomes impermeable to tracers around 4 dpf, while the midbrain does not become fully impermeable until 5 dpf [203]. This lapse in functional development can be attributed to lingering transcytosis [203]. The BBB in zebrafish continues to develop and is fully formed by 5–10 dpf [182,203].

Apart from zebrafish, there is little to no knowledge of piscine BBB development. Further studies should be conducted in order to identify whether variation exists in the development of glial barriers in elasmobranchs and sturgeons.

### 6.2. Function of the Piscine Blood-Brain Barrier

#### 6.2.1. Protective Barrier

The BBB of fish maintains the integrity of the central nervous system by strictly regulating the entrance of various compounds from the blood to the brain [10]. The BBB exhibits this defense through both active and passive mechanisms. Across fish taxa, the barrier has very low permeability to large molecular weight compounds. As previously mentioned, studies in several species have shown exclusion of compounds such as horseradish peroxidase, NHS sulfo-biotin, inulin, and Evan’s blue [2,178,188]. Early tracer studies were performed on goldfish, but high-molecular-weight compound exclusion was later performed in elasmobranchs, lamprey, hagfish, ratfish, and teleosts such as zebrafish [2,6,143,178,180,182,189,204]. Though structurally similar, several have concluded that the fish BBB is “leakier” than the mammalian BBB [2,143]. Additionally, an early study on the hagfish (*Myxine glutinosa*) led to the conclusion that they have a less effective and more permeable BBB; however, subsequent studies have demonstrated similar permeability to compounds such as (HRP) as mammals [5,188].

In addition to passive impermeability due to the presence of tight junctions, the BBB actively exports foreign compounds which are able to cross the BBB. ATP-binding cassette (ABC) transporters such as the xenobiotic export protein P-glycoprotein exist in several teleosts including zebrafish, killfish (*Fundulus herteroclitus*), and rainbow trout (*Onchornynchus mykiss*) [184,185,201,205,206]. P-glycoprotein and multi-drug resistant protein 2 are also found in isolated capillaries of the dogfish (*Squalus acanthias*) [205]. Although these proteins have been identified and functionally observed through inhibition of their activity, there is more knowledge to be gained regarding their specific activity at the BBB.

#### 6.2.2. Ion Transport and Enzyme Activity

Several levels of homeostatic control exist at the BBB in fish. Much like other vertebrates, several fish species regulate the balance of ions moving into and out of the brain parenchyma [178,207]. As neurons need a precise ionic environment to transmit signals, maintenance of ionic homeostasis is an essential function of the BBB [208]. This function has been identified in elasmobranchs in several species [178]. *Squalus acanthias*, *Raja batis*, and *Raja ocellata* regulate concentrations of Ca^2+^ and Mg^2+^, and *Squalus acanthias* may maintain K^+^ concentrations as well [178]. Ionic homeostasis is localized to glia in elasmobranchs, along with several other barrier properties [178]. Although ionic homeostasis is a well-described function of the BBB in mammals, very few studies have described this property in teleosts or conducted physiological studies. Further study is needed to determine what other similarities or differences exist between the ion transport of fish and other vertebrates, as well as whether there is variation across fish species.

The BBB is also a key regulator of metabolic function in the brain. This is because essential compounds such as glucose are regulated and transported across the BBB to enable proper brain function. GLUT-1 has been identified in the brain microvascular endothelial cells of zebrafish and rainbow trout [195,201,209]. GLUT-1 has also been identified not in the endothelial cells but in the perivascular glia of the Pacific sea shark (*Schroederichthys chiensis*) and the Mediterranean sea shark (*Scyliorhinus canicula*) [195]. Monocarboxylate transporters are also found in Pacific and Mediterranean sea sharks, predominately in the glial cells [195]. The localization of metabolic transport to the glia in elasmobranchs contrasts with other functions such as xenobiotic export, which are localized to the endothelium [205]. Interestingly, this indicates that some level of function in elasmobranchs is localized to the endothelium, while other functions are localized to the glial cells [205]. Apart from these studies characterizing key proteins, very few physiological studies have been performed in fish to determine a deeper barrier function. Additionally, it is uncertain whether sturgeons as well have barrier function localized to the glial level, since the structural barrier is at this level.

### 6.3. Factors Altering Piscine BBB Function

#### 6.3.1. Environmental Contaminants

As previously mentioned, xenobiotic efflux mechanisms exist at the BBB of many fish, but some environmental toxicants may cross the BBB and remain there at concentrations too high to export, or they may damage the BBB. Triclorfon and Aflatoxin B1 both cause increases in permeability of the BBB in silver catfish (*Rhamdia quelen*) [183,210]. These studies also found changes in behavior, though the link between BBB and behavior is uncertain for all fish [183,210]. Cadmium toxicity also causes increased permeability of the BBB in zebrafish, specifically by acting on scaffolding proteins securing tight junctions between endothelial cells [211].

#### 6.3.2. Microbial Infection

Several microbes may cause disruption of the integrity of the BBB. Specifically, the bacteria *Clostridium perfinges* produces Epsilon toxin, which increases BBB permeability in zebrafish [212]. Other bacteria such as *Mycobacterium tuberculosis* and *Mycobacterium marinum* can cross the BBB and reach the brain to cause infection [213]. In zebrafish, Group B *Streptococcus*, a leading cause of bacterial meningitis, breaks down tight junction proteins, thereby degrading BBB integrity in zebrafish [214]. Other microorganisms such as *Pseudomonas aeruginosa* elicit damage to the BBB in silver catfish by inducing the production of ROS [183].

#### 6.3.3. Nitric Oxide

Nitric oxide (NO) is a compound readily produced in biological systems by nitric oxide synthase [215,216]. NO is produced in several cell types, including those in the BBB [215]. In endothelial cells, NO acts as a signaling molecule initiating control of vasodilation in smooth muscle cells and permeability of the barrier [215,216,217]. Despite its biological importance, high levels of NO (from NO donor glyceryl trinitrate) caused increased permeability of the BBB in the common carp (*Cyprinus carpio* L.) for several hours before returning to its normal impermeability [215]. The BBB disruption by the innate compound NO highlights the importance of examining other compounds found within animals that when in imbalance may temporarily or chronically open or damage the BBB. Additionally, as previously mentioned, there is little information on mechanisms of disruption at the BBB in fish, indicating the need for further study and understanding in this area.

#### 6.3.4. Hyperosmotic Stress

At the glial barrier of the skate *Raja erinacea*, hyperosmotic stress caused by increased concentration of sodium chloride or fructose increased the permeability of the BBB to multiple compounds [218]. Further examination of this phenomenon is important, given the importance of ionic balance around the BBB [15].

### 6.4. Knowledge Gaps/Limitations

Though much is known so far regarding the BBB of fish, much is still unknown. Several studies have been conducted, but the species under examination are limited along with the regard for the ecological and evolutionary context of the organisms in question. We propose that it is essential to consider these factors more deeply in future studies and expand BBB studies to involve a broader understanding of the fish being studied. To begin to answer these questions, we first identify specific knowledge gaps among the fish:

- Does the increased neurogenesis found in fish impact the BBB?
- What undiscovered factors alter the BBB, and how do they alter function as well as form? Many studies thus far have focused mainly on how form is altered, but little is known about functional characteristics that may also be impacted.
- How does the life history of different fishes impact their BBB structure?
- Why do elasmobranchs and sturgeons have a glial BBB—what adaptive value does this provide?
- What are the mechanisms linking disruption of the BBB and behavior?
- How does the glial barrier seen in sturgeons and elasmobranchs develop compared to the endothelial barrier seen in other fish?

## 7. Blood-Brain Barriers of Non-Vertebrate Chordates—Cephalochordata and Tunicata

The lancelets (Cephalochordata) are of considerable interest in the study of the evolution of the blood-brain barrier given their pivotal phylogenetic position. However, very little is known of lancelets BBB form and function. An examination of cell junctions in the branchial chamber (pharynx), anterior and posterior intestine and nervous system epidermis and glial cells of *Branchiostoma lanceolatum* found no evidence of tight junctions in any tissue [219]. However, there were extensive desmosomal-like zonulae adhaerentes in neural epidermis and glial cells, and cadherins have been localized in *Branchiostoma belcheri* [220].

Several studies have investigated the nature of cell junctions in various tissue of the tunicates [221,222,223,224,225,226]. Tight junctions occur in the intestinal tract [226] and epidermis [227] of tunicates. Genomic analysis has also been employed to probe the molecular evolution of ion channels genes [228]. Functionally, the relationship between neuronal expression of Na^+^ and K^+^ channels and gap junctions in neuronal tissue has been explored [229]. Despite these studies, very little is known about the nature of cell–cell junctions in neuronal tissue of the non-vertebrate chordates. The nervous system of some tunicates can be as simples as ~100 neurons [230], yet presumably there is still some protection afforded neural tissue. However, no studies to our knowledge have been carried out on any putative blood-brain barrier equivalents.

## 8. The Blood-Brain Barrier of Non-Chordate Deuterostome Invertebrates

The non-chordate deuterostome invertebrates comprise the hemichordates and echinoderms. Unfortunately, as for the non-vertebrate chordates, almost nothing is known about the nature of the NVU in these animals, despite their pivotal phylogenetic position in relation to vertebrates. Structural characteristics of cell junctions have been determined [229,231,232], and connexins emerge as the major structural molecules of gap junction intercellular channels [231,233] Even fewer studies have determined the functional characteristics, though the paracrine regulation of ATP release as a modulator of Ca^2+^ wave transmission has been assessed in the gap junctions of deuterostomes. Gap junctions in deuterostomes (and protostomes) show permeability to small (<1 kDa) molecules and ions [234]. However, even though a thorough description of the complexity of the nervous system of non-chordate deuterostomes [235,236,237,238,239], some which have large, complex brains [240], has been achieved, and despite numerous accounts of the circulation in these taxa [241,242], investigation of the boundary between the vascular system and the nervous system is almost completely lacking.

## 9. The Blood-Brain Barrier of Protostome Invertebrates

In protostome invertebrates, both open and closed vascular systems occur, and in the case of open vascular systems, the circulating fluid is generally referred to as hemolymph. For the sake of both simplicity and continuity, however, we will informally refer to hemolymph as “blood” and refer to the interface between the hemolymph and the brain tissue as the blood-brain barrier.

The interfaces between hemolymph/blood and the CNS of protostome invertebrates are as diverse as these invertebrates themselves. Rotifera [243], Gnathostomulida [244], and Chaetognatha [244] possess only a thin sheath of extracellular matrix (ECM). More complex is the arrangement of ECM with underlying parenchymal glial cells in Platyhelminthes [244], Nemertea [245], Phoronida [244], and Onychophora [244], or a thin glial sheath in Nematoda [246]. Most complex multi-layered arrangements including vascular endothelia and/or glial epithelia are found in members of Mollusca and Arthropoda (see below). It is only within these latter two taxa that a BBB has been described ultrastructurally and functionally [21].

Importantly, the complexity of the circulatory system appears not to be correlated with the presence of a BBB. In Annelida, for instance, some taxa exhibit an elaborate vascular system which is often referred to as (semi-)closed [247]. However, only the major longitudinal vessels are lined with a continuous cellular wall, and the fine vessels (if this term is applicable at all) are lined by only a basal lamina [247,248,249]. No BBB has been found in members of Annelida [21,22,247,250,251]. Similarly, no BBB has been described in Xiphosura [252], despite the remarkable complexity of their circulatory system [253]. On the other hand, insects show a highly restrictive BBB [21] (see below) without the presence of an elaborate vasculature [254].

### 9.1. Arthropods

#### 9.1.1. Form

**Figure 4:**
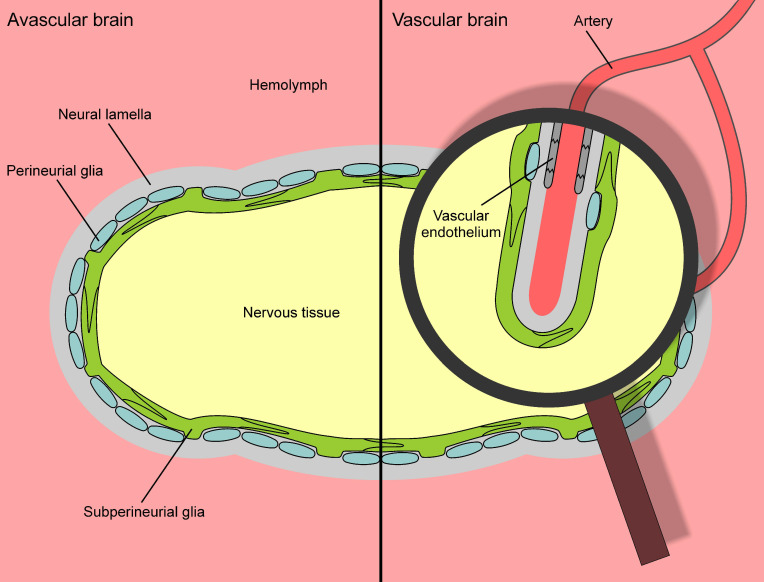
Schematic representation of the blood-brain barrier in arthropods. Cross section of an arthropod brain. Avascular supply of the brain (**left**) as found, e.g., in insects: The brain is surrounded by lacunae filled with hemolymph which bathes and thus supplies the brain. Vascular supply of the brain (**right**) as found, e.g., in decapod crustaceans and some arachnids (such as spiders and scorpions): arteries enter the brain and at their open endings release hemolymph into fine lacunae. A BBB exists between the nervous tissue and both the fine intracerebral lacunae as well as the lacunae surrounding the brain.

Mature arthropods. The blood supply to the brain in arthropods differs from that of vertebrates due to the open nature of the vascular system of arthropods. While there are arteries entering the brain in some arthropods [255,256], the hemolymph eventually leaves the vascular system. Exchange of substances between hemolymph and tissue occurs in the lacunar system, whereas circum- and intra-ganglionic vasculature (if present at all) mainly create sufficient hemolymph flow in the lacunae surrounding the supplied tissues [255,256,257,258,259]. Thus, the BBB in arthropods is not part of, nor connected to, the vascular walls. Rather, the BBB represents an interface between the brain tissue and the surrounding lacunae (Figure 4).

Blood-brain barriers can be found in species of all four major arthropod groups [260,261,262,263]. While exhibiting a certain degree of morphological disparity in regards to histological and ultrastructural details, the BBB in arthropods is generally made of at least the neural lamella, i.e., an outer sheath of connective tissue plus the perineurial glial epithelium. In insects, a layer of scattered perineurial glia is situated between the neural lamella and the epithelium. Consequently, the epithelium is referred to as subperineurial glia [262,264,265,266,267] (Figure 4).

The neural lamella, a prominent basement membrane, consists of collagenous fibers—characterized as collagen IV in *Drosophila* [262,268,269]—as well as mucopolysaccharides and proteoglycans [270,271,272]. While initially secreted mainly by hemocytes, the perineurial glia constantly act in maintenance and remodeling of the neural lamella [260,269,271].

The outer cell layer of the BBB in insects is a thin, discontinuous monolayer of perineurial glial cells [260,270,273,274]. The perineurium is not a closed layer because the underlying subperineurial glial cells also have contact with the neural lamella [266]. The cells of the perineurium are mainly connected via gap junctions [260,264].

The (sub)perineurial glial epithelium consists of remarkably voluminous polyploid cells [24] that show extensive interdigitations [260,261,262,264,266]. These complex interdigitations greatly increase the diffusion distance to be overcome [261].

In terms of intercellular junctions, several different conditions exist in the different main arthropod taxa [22]. In the dipteran insect *Drosophila melanogaster*, by far the most extensively studied arthropod in this context, pleated septate junctions are the dominant intercellular junctions. Given the lack of tight junctions in the subperineurium, these junctions mediate the barrier functions of the *Drosophila* BBB [262,264,275,276,277]. In the adult dipteran *Musca domestica* as well as in the blowfly *Calliphora erythrocephala*, coexistence of pleated septate junctions and punctate tight junctions has been described [278], as has been for the stick insect *Carausius morosus* [270,279]. However, in the hawkmoth *Manduca sexta*, only tight junctions occur [280]. Thus, *Drosophila* appears to be an exception, as in most other arthropods (such as other insects or arachnids) septate junctions are less frequent or even completely lacking in the BBB. Instead, tight junctions of different configurations (punctate, fasciar, or even zonular) provide an occluding function, as shown in, e.g., [22,278,280,281,282]. Myriapoda exhibit another kind of restrictive intercellular junction in the BBB–zonular linker junctions [22,283]. However, while the sheath around the brain exhibits a similar composition [263,284], little is known about the permeability of the putative BBB in myriapods.

In Xiphosura, the entire nervous system is situated within the arterial lumen [253]. The outer perineurium as well as the subperineurial epithelium are lacking and the nervous system is only lined by a thin neural lamella [252]. The parenchymal glial cells between the neurons show neither tight nor septate junctions [252] and do not constitute a restrictive BBB [252,285].

In short, elaborate BBBs are found in arachnids, insects, and some decapod crustaceans (e.g., crayfish). The glial sheath in marine crabs, however, is only partially restrictive and mostly delays diffusion [21,255], while perineurium and subperineurium are completely absent in horseshoe crabs [252]. This leads to the assumption that the hemolymph composition in marine environments is more stable than in freshwater and terrestrial habitats and the latter environment in particular calls for an effective BBB [252,286].

Developing arthropods. Development of the BBB has mainly been studied in insect larvae due to their ready accessibility, e.g., compared to larvae/juveniles of crustaceans. Development of the BBB in arthropods is so far best understood from the BBB morphology in the larval fruit fly *Drosophila melanogaster*. In this species, the subperineurial glial cells form a secondary epithelium via mesenchymal–epithelial transition. These cells originate from the ventrolateral neuroectoderm and migrate towards the outer margin of the central nervous system at about 17 h of development, with pleated septate junctions starting to form shortly after [287]. At the end of embryogenesis, the subperineurial epithelium is closed and the BBB established [264,288]. During postembryonic development, the integrity of the BBB is maintained by cessation of further cell proliferation. Instead, subperineurial cells become polyploid [289,290] and show extreme hypertrophy [264,291]. The early pleated septate junctions which form during establishment of the subperineurial epithelium are structured in a highly corrugated fashion, regulated by the G protein-coupled receptor (GPCR) Moody pathway, allowing for extensive stretching of the junctions during hypertrophy of the subperineurial glial cells [266,267]. While there is comprehensive knowledge about BBB formation in *Drosophila*, we emphasize that *Drosophila* might only give limited insight into insects/arthropods in general, because of the fruit fly’s more prominent role of septate junctions and the lack of tight junctions in the BBB (see section: Mature Arthropods).

In another dipteran, the blowfly *Calliphora erythrocephala*, the BBB is still leaky at the point of hatching, but becomes far more impermeable within the first few hours after hatching [292]. In larval stages, both pleated septate as well as tight junctions provide occlusion of the epithelium [292]. In early pupal stages, internal organs such as the CNS are reorganized, as is the BBB. Intercellular junctions in the BBB are resolved shortly after pupation and reappear in the reorganized BBB of the late pupal stages [292]. In late pupal as well as adult blowflies, both tight and septate junctions coexist in the BBB [292].

The hawk moth *Manduca sexta*, which does not exhibit septate junctions in any developmental stage, possesses a functioning BBB with vast punctate tight junctions already in late embryonic stages [280].

In the hemimetabolous stick insect *Carausius morosus*, the BBB is fully developed in newly hatched nymphs and until adulthood only changes in thickness. In *C. morosus*, both septate junctions as well as punctate tight junctions appear in the BBB [279].

#### 9.1.2. Function

Most functional analyses of the blood-brain barrier in arthropods were carried in the 1970s and 1980s as either transmission electron microscopical tracer analyses or electrophysiological experiments. The exception to this is mostly for research in *Drosophila*.

While the neural lamella may have the ability to restrict some larger molecules [274], ultrastructure tracer analyses show that the (sub)perineurial epithelium restricts penetration of ionic lanthanum in crayfish, arachnids, and insects [257,261,280,292]. All taxa reveal similar forms of diffusion restriction through the intercellular clefts via occluding junctions (septate and/or tight junctions, see above).

Electrophysiological experiments have been restricted to larger and thus more accessible arthropods, such as crayfish. Electrophysiological experiments in the nervous system of *Procambarus clarkii* have shown a high permeability of the perineurial epithelium for K^+^ and virtual impermeability to Na^+^ [293]. Further investigation found a high Ca^2+^-dependence of K^+^ permeability caused by voltage-gated Ca^2+^ channels and Ca^2+^-dependent K^+^ channels, highlighting the important role of transcellular transport mechanisms in ion regulation in the crayfish [294]. The high transcellular K^+^ permeability (10 times higher than the Cl^−^ permeability) and a lower paracellular K^+^ permeability allows for the maintenance of a low interstitial concentration of K^+^ to aid neurological function [293,294]. In the cockroach, *Periplaneta americana*, a constant efflux of K^+^ from the brain is maintained in different external K^+^ concentrations [295]. While ion homeostasis is in large part provided by activity of the Na^+^/K^+^-ATPase [296], ATP-binding cassette and solute carrier transporters are extensively expressed in the *Drosophila* BBB, where they likely play an important role in chemoprotection as well as nutrient supply [26]. While expression of transport proteins in the subneurial epithelium of *Drosophila* occurs, their actual physiological function is yet to be studied [265].

#### 9.1.3. Factors Altering Structure and Function

Numerous factors potentially affect the integrity of the arthropod blood-brain barrier. In the cockroach, *Periplaneta americana*, treatment with urea reduces the net K^+^ efflux to the extent where there is more or less no net flux, likely due to increased paracellular permeability. In contrast, the antibiotic Amphotericin B significantly increases K^+^ efflux from the brain by increasing transcellular permeability [295]. Treatment of the cockroach BBB with the neuroactive hormone octopamine greatly reduces K^+^ permeability of the BBB and allows for the assumption of potential hormonal control of BBB permeability [297]. Treatment of the cockroach CNS with the K^+^ channel blocker Ba^2+^ significantly reduces K^+^ efflux from the ganglion, whereas the Na^+^/K^+^-ATPase inhibitor ouabain leads to a large increase in the K^+^ efflux from the brain [295].

In *Drosophila*, the temperature sensitivity and effect of cold adaptation on the Na^+^/K^+^-ATPase was recently studied [296]. Cold-induced changes in transcellular permeability appear to be accompanied by altered paracellular permeability as well, the complex interaction of which is yet to be studied. In general, endocytosis activity in the BBB differs between sleep and wake periods, and blocking endocytosis in the BBB of *Drosophila* increases sleep periods [298]. Using the xenobiotic rhodamine b, the study of the effect of sleep on the BBB in *Drosophila* reveals a circadian rhythm of BBB permeability [299]. Higher BBB permeability during the nighttime allows for higher effectiveness of some neuroactive drugs when applied during the night, as also shown for the anti-epileptic phenytoin [298,299].

Recent research on Carniolan honeybees (*Apis mellifera carnica*) showed ontogenetic and starvation-induced alterations of metabolism in neuroglia. Both aging and starvation led to a decrease in the amount of the metabolic enzyme glycogen phosphorylase in the BBB [300]. In *Drosophila*, starvation leads to an increase in expression of the carbohydrate transporter Tret1-1 to maintain a prioritized nutrient supply of the brain in times of insufficiency [301].

### 9.2. Molluscs

#### 9.2.1. Form

Members of most subtaxa of Mollusca exhibit an open hemolymph vascular system, wherein hemolymph leaves the arteries, is further channeled through lacunae as part of the hemocoel, and then ultimately collects in veins afferent to the central heart [302]. Only Cephalopoda have evolved a closed blood vascular system with direct transition from arteries to veins via capillary networks [302]. The true vessels in molluscs are lined with an endothelial wall and the blood pressures of some molluscs (cephalopods in particular) are comparable to those of some vertebrates [302,303]. With varying degrees of “openness”, there also exists morphological variation of the CNS blood supply: the CNS presents avascular supply being bathed in hemolymph in bivalves [304] and aquatic gastropods [305], a supply via fine vessels and/or widened vascular hemolymph spaces in terrestrial snails [306,307], and vascular supply via true capillaries in cephalopods [308,309]. Thus, a vascular endothelium at the interface between the CNS and the blood can be absent or present depending upon taxon within the Mollusca.

**Figure 5:**
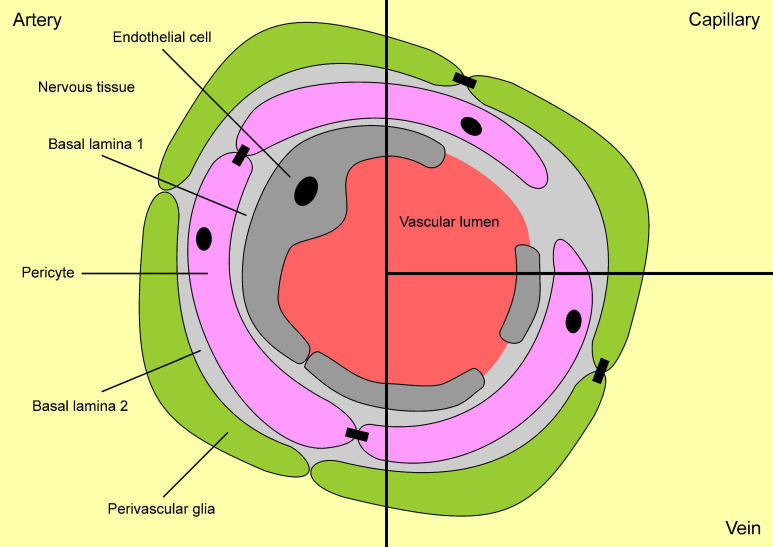
Schematic representation of the blood-brain barrier in cephalopods. Cross section of vessels in the brain; black bars indicate the site of the BBB. In arteries, the vascular endothelium is continuous; the diffusion barrier is located at the layer of pericytes. In capillaries and veins, the vascular endothelium is discontinuous; the diffusion barrier is located at the level of the perivascular glial epithelium. The vascular endothelium in capillaries is highly discontinuous; in the finest capillaries, the pericyte layer is discontinuous as well.

Of all molluscs, the BBB is best studied in cephalopods, especially in the cuttlefish *Sepia officinalis*. The brain of *S. officinalis* is supplied by numerous fine arteries that ramify into capillaries, which in turn unite to form veins leaving the brain [308]. The ultrastructure of the arteries inside the brain differs from that of capillaries and veins and so does the localization of the BBB [208,310] (Figure 5). The interface of the artery and brain consists of (1) the continuous vascular endothelium, (2) a first basal lamina, (3) a continuous layer of pericytes, (4) a second basal lamina, and (5) a continuous layer of perivascular glial cells [18,308,310]. In contrast, in capillaries and veins, the endothelium is discontinuous. Consequently, in regions where no endothelium is present, there is only a single basal lamina [308,310]. In the smallest capillaries, the pericyte layer may also be discontinuous [310] (Figure 5). The perivascular glial cells do not interdigitate extensively but rather show a large overlap that extends the intercellular cleft; thus, the distance over which materials must diffuse to overcome [310]. There are no tight junctions in any of the cellular layers [308]. Cell occlusion is provided by linker junctions in the pericyte layer of arteries and the perivascular glia of capillaries and veins [18,22,208,308,310]. Tracer experiments show that in arteries, diffusion is restricted by the pericytes, whereas the perivascular glia is the restrictive layer in capillaries and veins. Essentially, this means that two different kinds of BBB coexist in *S. officinalis* [18].

In gastropods, the central nervous system is surrounded by a capsule of connective tissue. In the terrestrial snail *Helix pomatia*, this sheath is highly vascularized, and the small vessels widen to form the vast periganglionic vascular space, while the ganglion itself is avascular [306,311]. The interface between hemolymph and the neurons consists of two or three layers: (1) the vascular endothelium (which is absent in at least the aquatic gastropods *Viviparus contectus* and *Lymnaea stagnalis*; [305]), (2) a thick layer of extracellular matrix, predominantly consisting of collagen fibers, and (3) a monolayer of perineurial glial cells with interdigitating processes [305,306,307,312]. In *Helix pomatia* the endothelium only restricts diffusion of larger particles, such as colloidal carbon (50 nm); [306]. The ECM layer and the glial perineurium are putative sites of the BBB in gastropods. While there are some descriptions of the ultrastructure of the glial layer in gastropods, e.g., [305,306,307,311,313,314,315,316], little is known about the presence and distribution of intercellular junctions. While single punctate tight junctions have been reported for the terrestrial snail *Helix aspersa* [314], a lack of (semi-) restrictive junctions occurs in the perineurial glia in gastropods in general. Gap junctions as well as desmosomes appear to be the dominating types of junctions [22,281].

Little is known about the sheath around the CNS in bivalves. The CNS is coated by a neural lamella and there is no perineurial layer of glial cells. Instead, parenchymal glial cells are situated between the neurons, as shown for *Anodonta cygnea* [304]. There are no (semi-)restrictive junctions reported for bivalves and a BBB is most likely absent in bivalves in general [22].

#### 9.2.2. Function

The function of the molluscan BBB has been studied in a few studies using tracer experiments and electrophysiological assessment of ion fluxes across the periganglionic glial sheath.

The cephalopod *Sepia officinalis* exhibits a BBB that is largely impermeable to a range of molecules, from small to large (horseradish peroxidase, polyetholene glycol, and EDTA) as well as to lanthanum ions [18,309]. All restriction of diffusion in the intraganglionic arteries happens at the layer of the vascular pericytes because no tracer is found at the second basal lamina [18]. In capillaries and veins, horseradish peroxidase as well as lanthanum pass the pericytes and second basal lamina but are blocked by the perivascular glial sheath [18]. This presents the unique condition that two different BBB mechanisms coexist in the very same animal, as described above.

In the terrestrial snail *Megalobulimus abbreviates*, lanthanum does not pass through the perineurial glial sheath [307]. Electrophysiological assessment of ion fluxes in the abdominal ganglia of *Helix aspersa* shows differential potassium flux in de-sheathed and intact ganglia, pointing towards some K^+^ restriction due to the perineurial glia, which might be even stronger in the brain [317]. In contrast, in the aquatic snails *Viviparus contectus* and *Lymnaea stagnalis*, no difference in ion exchange between the de-sheathed and intact brain was discovered, indicating the absence of a BBB [305,318].

### 9.3. Knowledge Gaps/Limitations for Invertebrate Blood-Brain Barriers

Recent research of blood-brain barriers in invertebrates is mostly restricted to *Drosophila*, although honeybees appear to be of increasing interest in BBB research. With the recent findings in insects, the impact of BBB activity and possible alterations of BBB function on insect behavior has emerged as a perspective field of research [300,319]. The focus on insects, however, has led to a neglect of other arthropod taxa; thus, new insights from a comparative approach on different arthropod groups are not in sight. The same holds true for molluscs, wherein—to the best of our knowledge—no recent research on the BBB exists. Further interesting questions for future research of BBBs in invertebrates include:

- Are there differences in BBB function between arthropod taxa with tight junctions in their (sub)perineurium (e.g., the emerging spider model *Parasteatoda tepidariorum*) and those without (e.g., *Drosophila*)?
- What is the influence of terrestriality on presence and tightness of the BBB in invertebrates (e.g., aquatic vs. terrestrial snails; marine vs. freshwater vs. terrestrial decapods; aquatic insect larvae vs. terrestrial adults)?
- Can differences in salinity and thus hemolymph osmolarity be correlated with differences in BBB tightness in closely related species (e.g., lobsters vs. crayfish), thus pointing towards evolutionary causes?
- What is the influence of higher sensory input on presence and tightness of the BBB (e.g., comparison within insects, crustaceans, or cephalopods)?
- Are there trends towards convergent evolution of BBB function in different groups of eusocial insects?
- Does toxicological alteration of BBB permeability affect complex behavioral patterns in invertebrates (e.g., courtship behavior in scorpions or the cephalopod *Sepia officinalis*)?

## 10. Conclusions and Future Directions

### 10.1. The Plurality of Blood-Brain Barriers

The need to protect neural tissue from toxins or other substances that could disrupt neural function is as old as neural tissue itself. Not surprisingly, then, blood-brain barriers (or their equivalent) can be found in almost all animals with discrete neural tissue and complex sensory/motor ability. From a functional perspective, the role of establishing a barrier between vascular spaces and neural tissues is consistent across vertebrates and invertebrates; how this is achieved morphologically is not, there being a variety of morphological arrangements achieving neural tissue protection (Figure 1, Figure 2, Figure 3, Figure 4 and Figure 5). Of note, then, is that blood-brain barriers have independently evolved multiple times. Indeed, it is simplistic to base comparative morphological or evolutionary studies of the BBB on the view that the vertebrate BBB (itself not consistently an endothelial barrier across taxa) derived from “the invertebrate BBB”. Indeed, the term blood-brain barrier is heavily slanted towards function, whereas, morphologically, we should use the plural form of ‘barriers’ to correctly imply the differences observed in structures. Put differently, the BBB across broad taxa is an analogous, not homologous, assemblage.

### 10.2. Areas for Future Research

Despite more than a century of research on the characteristics of blood-brain barriers, there remain many poorly explored aspects of invertebrate and vertebrate blood-brain barriers.

#### 10.2.1. Environment and the Evolution of the Blood-Brain Barrier

How the environment shapes the blood-brain barrier, especially in the selection pressures leading to an animal’s evolution, has remained somewhat enigmatic. However, understanding the selective pressure acting on the barriers protecting neural tissue is key to any evolutionary interpretation and subsequent extension to the mammalian condition. For example, does the variable blood/hemolymph ion concentrations of osmoconformers translate into different characteristics of the blood-brain barrier, or have the lifestyle characteristic of intermittent feeding (e.g., snakes that eat large amounts but only infrequently) and the attendant large swings in blood glucose levels resulted in modification of blood-brain barrier transport functions over evolutionary time?

#### 10.2.2. Plasticity of the Blood-Brain Barrier

Another aspect of blood-brain barriers that deserves more attention involves the plasticity of these blood-brain barriers—both morphologically and physiologically. The concept of plasticity of course spans both developing animals and established adults. Research has been performed on the development of the blood-brain barrier in the zebrafish and the fruit fly (which, as mentioned above, are not necessarily broadly representative of teleosts and insects, respectively, and so may not be the best model for extrapolation to mammals) and other animal models. However, the overall malleability by internal and external environmental factors during development and subsequently in the adult itself is still not clear. For example, can changes in BBB form and function induced in the developing animal persist into adulthood, or does the BBB revert to its typical form in the adult? That is, are changes induced during development transient, or do they ‘stick’ to the adult? Are these changes in BBB form and function reversible by a change in internal or external environment?

#### 10.2.3. The Blood-Brain Barrier and Behavior

Perhaps one of the most fundamental unanswered questions is: how do both the innate characteristics and induced changes in the blood-brain barrier affect an animal’s behavior? While numerous studies have shown both in vitro and in vivo changes in barrier function, causal studies of how such BBB changes might result in subtle or overt changes in behavior are largely lacking.

It is frequently said that the study of the brain is the ‘last frontier’ in science. If so, and continuing the frontier analogy, then the blood-brain barrier is the critical border between civilization and largely uncharted new territories of the frontier.

## Figures and Tables

**Table 1:** Differences in the neurovascular unit of fish.

Group	Main Barrier Location	Endothelial Cells	Glial Cells	Pericytes	Basal Lamina	Other Notes	References
Teleosts	Endothelium	Present	Present, Radial glia	Present	Present		Jurisch-Yaksi et al., 2020; Jeong et al., 2008; Fleming et al., 2013; O’Brown et al., 2018
Sturgeons	Glia	Present, overlapping with tight junctions, leaky due to caveolae and vesicles	Present, not joined by tight junctions, possibly gap junctions/interdigitating glial lamellae	None	Present	Endothelium not as leaky as in elasmobranchs	Bundgaard and Abbott, 2008
Lungfish	Endothelium	Present, joined by tight junctions	Present, discontinuous	Undetermined/“Perivascular cells” rather than glial cells	Reduced, not present on brain side	Discontinuous glia allow neurons to interact with pericytes, basal lamina, endothelium	Bundgaard and Abbott, 2008
Bichirs	Endothelium	Present, continuous tight junctions	Present, discontinuous	Undetermined	Reduced		Bundgaard and Abbott, 2008
Lamprey and Hagfish	Endothelium	Present, joined by tight junctions, overlapping	Present, continuous sheath	None	Present	Endothelial cells have many abluminal vesicles and tubules	Bundgaard, 1982; Bundgaard et al., 1979
Chimaera	Endothelium	Present, described as “thick”	Present, discontinuous	None	Present, possibly covering glial processes as well		Bundgaard, 1982
Elasmobranchs	Glia	Present, leaky, and not joined by tight junctions	Present, radial glia, continuous, joined by tight junctions	Present	Present		Bundgaard and Cserr, 1981; Balmaceda-Aguilera et al., 2012; O’Brown et al., 2018

## Data Availability

Not applicable.
